# Supplementary material for: Gene polymorphisms of Patatin-like phospholipase domain containing 3 (PNPLA3), adiponectin, leptin in diabetic obese patients
Source: PLoS One. 2020 Jun 16;15(6):e0234465. doi: 10.1371/journal.pone.0234465 (PMC7297308; doi:10.1371/journal.pone.0234465)
Supplement: Attachment — Submitted filename: Dear.pdf [file pone.0234465.s002.pdf]

Dear
English language needs revision and paragraphs need rephrasing as well
More details are required in the methods
Limitations and future prospective are to be there
Good luck
