# Supplementary material for: Gene polymorphisms of Patatin-like phospholipase domain containing 3 (PNPLA3), adiponectin, leptin in diabetic obese patients
Source: PLoS One. 2020 Jun 16;15(6):e0234465. doi: 10.1371/journal.pone.0234465 (PMC7297308; doi:10.1371/journal.pone.0234465)
Supplement: Attachment — Submitted filename: Respnse to reviewers.docx [file pone.0234465.s003.docx]

**Gene Polymorphisms of Patatin-like phospholipase domain containing 3 (PNPLA3), Adiponectin, Leptin in Diabetic Obese Patients**

1. **Comment: ʺEnglish language needs revision and paragraphs need rephrasing as wellʺ**

**Response: the whole manuscript starting from the title was revised by English native speaker and many paragraphs were rephrased or almost rewritten.**

2. **Comment: ʺMore details are required in the methodsʺ**

**Response: each protocol for each parameter was written as a separate item with separate title and the details for each technique was added including principle, steps, kit manufacturer and the machine used.**

3. **Comment: ʺLimitations are to be thereʺ**

**Response: a title of (Limitations of the study) was added after the conclusion section and all expected limitations were mentioned under this section.**

4. **Comment: ʺFuture prospective are to be thereʺ**

**Response: a title of (Future Prospective) was added after the limitations of the study section and all recommended future work was mentioned under this section.**

***All changes done in the manuscript according to reviewer comments are highlighted in yellow and English language and grammar revisions are done but not highlighted as they are frequent.**
