# Supplementary material for: Gene polymorphisms of Patatin-like phospholipase domain containing 3 (PNPLA3), adiponectin, leptin in diabetic obese patients
Source: PLoS One. 2020 Jun 16;15(6):e0234465. doi: 10.1371/journal.pone.0234465 (PMC7297308; doi:10.1371/journal.pone.0234465)
Supplement: Attachment — Submitted filename: REPLY.docx [file pone.0234465.s004.docx]

1. English editing is highly recommended throughout the manuscript.
2. Abstract:

- Rephrase line 35-37.

3. Introduction:

- Add more references on PNPLA3, lane 76

4. Methods:

- “Fresh venous blood samples” explain what do you mean by “fresh” this is not an accurate word here, explain?? And rephase.
- Add references for PNPLA3 amplification and genotyping sections.
- A figure for PNPLA3 results should be added.

5. Results:

- include a table with the demographic data of all participants.
- Rephrase lines from 190-194 to be more simple and comprehensive.

6. Discussion:

- Authors should compare results of genotypes distribution of the genes with other studies in Egypt, specially in controls.
- Authors should compare polymorphism results of all genes with those of other studies.

7. Limitations of the study:

- State clearly that the small sample numbers is a limitation in the study of polymorphism of these genes in the herein study.
