# Supplementary material for: Gene polymorphisms of Patatin-like phospholipase domain containing 3 (PNPLA3), adiponectin, leptin in diabetic obese patients
Source: PLoS One. 2020 Jun 16;15(6):e0234465. doi: 10.1371/journal.pone.0234465 (PMC7297308; doi:10.1371/journal.pone.0234465)
Supplement: Attachment — Submitted filename: Respnse to reviewers.docx [file pone.0234465.s005.docx]

**Response to Reviewers**

**Gene Polymorphisms of Patatin-like phospholipase domain containing 3 (PNPLA3), Adiponectin, Leptin in Diabetic Obese Patients**

1. **Comment:** English editing is highly recommended throughout the manuscript **ʺ**

**Response: the whole manuscript was revised.**

2. **Comment: ʺ**Abstract: Rephrase line 35-37**ʺ**

**Response: lines 34-37 were rephrased.**

3. **Comment: ʺ**Introduction: Add more references on PNPLA3, line 76**ʺ**

**Response: 3 more references were added, lines 78, 79.**

4. **Comment: ʺ Methods: Fresh venous blood samples” explain what do you mean by “fresh” this is not an accurate word here, explain?? And rephraseʺ**

**Response: it was written by mistake and it is modified into five ml**

5. **Comment: ʺ Methods: “Add references for PNPLA3 amplification and genotyping sections. ʺ**

**Response: the reference is added (Dutta, 2011) line 128**

6. **Comment: ʺ Methods: “A figure for PNPLA3 results should be added.ʺ**

**Response: the gel electrophoresis pictures were added (figure 1 for PCR product at line 134) and (figure 2 for genotying, line 153).**

7. **Comment: ʺResults: include a table with the demographic data of all participants ʺ**

**Response: We put it at the supporting information files.**

8. **Comment: ʺResults: Rephrase lines from 190-194 to be more simple and comprehensive ʺ**

**Response: most of results paragraphs were made simpler and more comprehensive**

9. **Comment: ʺDiscussion: Authors should compare results of genotypes distribution of the genes with other studies in Egypt, especially in controls.**

**Response: for PNPLA3 genotyping there is only one study was done in Egypt which was on Hepatitis C infection and liver fibrosis. We added citation for this study in discussion section with comparison between control groups of this study and our study. So this is the first time to assess PNPLA3 genotype frequency in diabetic obese patients in Egypt. ADIPOQ and LEP gene polymorphism which were done in Egypt were targeting other sites other than our target on ADIPOQ rs266729 and Leptin rs2167270.**

10. **Comment: ʺ Discussion: Authors should compare polymorphism results of all genes with those of other studies ʺ**

**Response: Regarding PNPLA3 genotyping, we compared among our study and other different studies but there are very few studies which performed this polymorphism in diabetic obese patients but we mentioned them in discussion. Regarding ADIPOQ and LEP genotyping, were compared with different mentioned studies in the discussion with new citations.**

11. **Comment: ʺLimitations of the study: State clearly that the small sample numbers is a limitation in the study of polymorphism of these genes in the herein studyʺ**

**Response: the small sample size limitation is stated and recommendations were restated.**

***All changes done in the manuscript according to reviewer comments are highlighted in blue and English language and grammar revisions are done.**
